# Foreign Direct Investment’s Impact on China’s Economic Growth, Technological Innovation and Pollution

**DOI:** 10.3390/ijerph18062839

**Published:** 2021-03-10

**Authors:** Shihong Zeng, Ya Zhou

**Affiliations:** 1 Department of Applied Financial Economics, College of Economics & Management, Beijing University of Technology, Beijing 100124, China; 2 Finance and Economics Development Research Center, College of Economics & Management, Beijing University of Technology, Beijing 100124, China

**Keywords:** FDI, economic growth, innovation, pollution, dynamic panel simultaneous equation model

## Abstract

In recent years, China has gradually become one of the countries with the largest levels of foreign direct investment (FDI). FDI has played a significant role in promoting Chinese economic development, and the FDI technology spillover effect is one of the core forces driving China towards reaching new growth milestones. Therefore, due to the country’s interest in development, there is competition for FDI throughout China. However, the existing imperfect environmental protection system cannot prevent FDI from flowing into China’s highly polluting and resource-intensive industrial chain, possibly causing serious environmental problems. Therefore, the topic of properly introducing foreign capital to promote development and effectively end China’s current environmental pollution crisis has become a research focus. To explore FDI’s impact on China’s economic growth, technological innovation, and environmental pollution, we use Chinese provincial panel data for 2004–2016 to construct a dynamic panel simultaneous-equation model. Considering the interrelationships between the equations, we construct economic models of economic growth, technological innovation, and pollution emissions, and incorporate them into the same research system for generalized method of moments (GMM) estimation. Our results show that FDI has a significant and positive direct impact on China’s economic growth and technological innovation, and can furthermore have a significant pull effect on the domestic economy through the backward spillover channel. At the same time, FDI has a direct and significant impact on the increase in regional waste-water discharge, while exhibiting a pollution halo effect on the emission of sulfur dioxide (SO_2_) and chemical oxygen demand (COD) emissions directly. In addition, we observe “benign feedback mechanism” between technological innovation output and these three types of pollution, namely SO_2_ emission, COD emissions, and ammonia and nitrogen discharge.

## 1. Introduction

In recent years, China’s foreign direct investment (FDI) has reached 941.52 billion yuan, ranking among the highest worldwide. Since the start of reforms and opening-up, FDI has played an important role in promoting Chinese economic development [1], and, especially, the part of fostering technological innovation, which can facilitate the attainment of new growth milestones. As is well-known, the driving force and source of ensuring the sustainable development of a country’s economy is the country’s technological innovation capability, which plays an irreplaceable role in economic development [2,3,4]. Several studies have shown that China’s technological innovation can be enhanced by the international technology overflow effect via attracting FDI to “introduce–absorb–redigest” advanced technologies from developed countries and “standing on the predecessor’s shoulders” to innovate and then achieve the goal of improving the level of economic development [5].

However, while FDI brings economic growth, there may be potential hazards, the most obvious being environmental pollution. Due to the insufficiency of regional economic development and the existence of loopholes in the introduction of foreign capital, FDI tends to flow to energy-intensive and high-pollution industries. Therefore, an incalculable environmental cost has been incurred alongside regional economic growth [6,7,8,9].

Developed countries that have implemented advanced scientific and technological reforms and achieved high levels of economic development tend to move their high-pollution and energy-intensive industries to less developed countries. The research of Lin, B. and Xu, M. [10] indicates that China has gradually become a “pollution haven” in Sino-Russian trade. Cheng et al. [11] also find evidence to confirm the pollution haven hypothesis in China. To achieve temporary economic growth and improve employment rates, the governments in such less-developed countries and regions have implemented a series of measures to attract foreign capital at any cost and have continuously lowered the standards of environmental control. Li et al. [12] found that FDI companies tend to avoid areas with strict environmental regulations. To et al. [13] found that the pollution paradise hypothesis is generally valid in Asian emerging markets. The problem of environmental pollution has then arisen over time along with the inflow of capital and technology. With the emergence of global climate change, ozone layer depletion, water pollution, and other issues, the awareness of the “green” economic development in countries around the world has gradually strengthened.

There are multiple influences among FDI, economic growth, technological innovation, and environmental pollution. Only when the decision-makers have mastered all of the various impacts brought by FDI inflows can they effectively adjust the FDI’s introduction structure to achieve the goal of maintaining the standards of environmental protection and avoiding an extensive development mode while improving the level of technological innovation and ultimately promoting a healthy development of the domestic economy. Therefore, studying how to balance the relationships among FDI inflows, economic growth, and environmental protection and how to improve the innovation capacity of China’s industries is of great practical significance.

The purpose of this paper is to examine 30 provinces, municipalities, and autonomous regions (except Tibet) in China as the research object, comprehensively consider economic growth, technological innovation, and environmental pollution, and thoroughly analyze the impact of FDI inflows on various regions of China to provide scientific guidance to policymakers for formulating reasonable measures for the introduction of foreign capital. The contribution of this paper is to put FDI, economic growth, technological innovation, and environmental pollution into the same system model for study. Moreover, we construct a formula for calculating the FDI spillover effect at the provincial level in China, and show that FDI has a significant and positive direct impact on China’s economic growth and technological innovation. Furthermore, FDI can have a significant pull effect on the domestic economy through the backward spillover channel. In particular, FDI has a direct and significant impact on the increase in regional wastewater discharge while exhibiting a pollution halo effect on the emission of SO_2_ and chemical oxygen demand (COD) emissions directly. In addition, we observe “benign feedback mechanism” between technological innovation output and these three types of pollution, namely SO_2_ emission, COD emissions, and ammonia and nitrogen discharge. Therefore, this paper tries to contribute to the existing literature in several dimensions. First, we extend the Cobb-Douglas (C-D) function model by decomposing the innovation factor, following the research of Jerzmanowski [14], Zhang and Liu [15], and Klenow and Rodriguez-Clare [16], and then use a dynamic panel simultaneous equation model to include FDI, technological innovation, economic growth, and environmental pollution in the same research system, fully consider the internal links between the four, and then further analyze the impact of FDI. Second, we do not combine multiple pollution variables into a new index, as some previous studies have done, and instead add different types of pollution separately into the model to clearly identify the types of pollution that are more susceptible to FDI. Finally, we establish the indicators of FDI spillover effect at the provincial level in China, including horizontal, forward, and backward spillover effects. Those spillover effect indicators are established by following the method proposed by Javorcik [17], but our approach is different from the author’s calculation method based on micro-enterprises. We design the calculation formula from the regional level.

The remainder of the paper is organized as follows. In Section 2, the related research is summarized and analyzed. Section 3 introduces model construction and describes data. Section 4 presents the process of empirical testing and analyzes the estimation results. Section 5 provides the conclusions.

## 2. Literature Review

Scholars have studied the influence of FDI from many perspectives. The inflow of FDI can not only bring abundant investment capital to a country but also enable the country to enjoy its technological externalities to improve the technological development capacity of enterprises. In particular, the technology spillover effect of FDI is more apparent when foreign investors enter by means of technology or joint ventures. Considering rapid economic development, some scholars have begun to realize the negative impact of FDI inflows. In various economies, such impacts differ significantly in certain ways, and as environmental problems continue to erupt, governments are increasingly cautious about the introduction of foreign capital. In the following section, we present a detailed review of FDI’s impact on economic growth, innovation, and environmental pollution.

### 2.1. FDI’s Impact on Economic Growth

FDI mainly affects a country’s economic growth in two ways. First, FDI provides host countries with high-quality capital and stimulates them to expand investment and production to achieve economic growth. Second, the advanced production technology and management experience brought by FDI drive the host country’s enterprises to optimize their production efficiency, thus also facilitating economic growth [18]. However, according to studies, the results are sometimes not as optimistic as expected.

The new economic growth theory proposed by Romer [19] and Lucas [20] was the first to prove that investment can lead to economic growth. Ahmed [21] analyzed the spillover effect of FDI inflows using a time series regression model, proving that FDI inflows had a driving effect on production input, thus causing the growth of the Malaysian economy. Lee [22] considered G20 country data for 1971–2009 and used a panel data cointegration regression model to verify the positive direct impact of FDI on the economic growth of G20 countries. Domestically, Wang and Wang [23] performed a comparative analysis of foreign and domestic mergers and acquisitions in China, concluding that foreign ownership did not drive production but boosted China’s development by improving its financial position. Hong [24] and Peng et al. [25] considered Chinese provincial and municipal panel data. The former used the generalized method of moments (GMM) method to re-estimate the positive interaction between FDI and China’s production input, which supported China’s economic growth; the latter studied the relationship between economic growth and FDI by using a bootstrap Granger panel causality approach and concluded that causality between FDI and economic growth was not identical completely in different regions of China, being unidirectional or bidirectional. Considering environmental protection, Yue et al. [26] used a slacks-based measure and directional distance function (SBM-DDF) approach to build a model to analyze the economic growth efficiency of 104 cities in China. The results showed that, overall, FDI had a positive impact on China’s economic growth efficiency; however, the degree of impact varied by city and industry. Comes et al. [27] studied the relationship between economic growth and FDI by using panel data on seven central and eastern European countries with a low per-capita GDP, also concluding that FDI promoted economic growth. Orlic et al. [28] researched the spillover effects of foreign firms on the total factor productivity (TFP) of local manufacturing firms by using a dynamic panel model, and revealed that local manufacturing firms benefit from the presence of foreign firms in upstream knowledge-intensive services, and in the downstream manufacturing sector.

However, some scholars’ results showed that FDI inflows had no significant economic spillover effect on some countries and regions. For example, Belloumi [29] used an autoregressive distributed lag (ARDL) model to analyze the relationship between FDI and economic growth in Tunisia, determining that FDI in the country could not generate positive externalities for economic growth and even that there was no significant causal relationship between them.

In summary, one can easily conclude that FDI has positive spillover effects on economic growth. However, this finding has not been verified by Belloumi. Does this discrepancy mean that the economic development stages vary, and so do the results? Although the former has conducted a relevant study of Chinese regions and believes that FDI’s impact on China’s economic growth is positive, studying whether this result has by now changed due to rapid economic development is warranted.

### 2.2. FDI’s Impact on Technological Innovation

There are two ways for a country to realize technological innovation, namely independent innovation and technology introduction. Foreign-invested enterprises will bring some advanced production technology and management experience to the host country; therefore, FDI inflows may serve the purpose of technology introduction. On the other hand, the entry of foreign capital will further increase the competitiveness of the host country’s market. However, if local enterprises wish to compete with foreign enterprises with many advantages, the only choice of the former is to increase investment in research and development (R&D) to improve technical efficiency. Therefore, FDI to some extent stimulates the host country’s awareness of the need for independent innovation. However, another possible result is that due to the entry of FDI, the host country becomes overly dependent on the technology spillover effect of foreign capital; alternatively, it may remain merely at the technology replication stage with independent innovation consciousness being inhibited, leading to a decline in the innovation level. Thus, FDI’s impact on the innovation capacity of host countries is intricate and complex, and can mainly be of three types based on inhibition theory, promotion theory, and dual role theory.

Kemeny [30] selected 119 sample countries across Europe, the Americas, and Asia and constructed regression models with unbalanced panel data. The study observed that FDI was positively correlated with a country’s technological upgrading; however, this effect was limited by the country’s initial level of technological development and social income capacity. The richer the country’s economy was, the weaker the technological spillover effect of FDI. Erdal and Göçer [31] focused on ten developing Asian countries and used a panel causality model and a cointegration model to analyze the FDI’s impact on R&D and innovation. The results showed that FDI inflows improved the R&D capacity of each country. Zhang [32] constructed Poisson and negative binomial models using Chinese provincial panel data for 2004–2012, finding that the spillover effect of foreign investment inflows had a positive impact on China’s overall innovation; however, there were differences in innovation efficiency in different regions. Wang and Wu [33] considered Chinese electronics companies and concluded that local innovations by foreign-funded enterprises could significantly facilitate product innovation of domestic firms; additionally, geographical proximity affected the technology spillover effect.

However, some scholars have questioned this market-for-technology strategy. García et al. [34] analyzed Spanish manufacturing enterprise data at the micro level and observed that FDI into Spain was negatively correlated with the innovation performance of local enterprises. Sasidharan and Kathuria [35] used panel data on Indian manufacturing enterprises to study the relationship between FDI and corporate R&D under India’s post-liberalization system. The results showed that the full sample’s analysis could not clearly explain FDI’s impact on the enterprises’ domestic innovation; however, an analysis of a subsample showed that FDI and R&D investment were complementary. The research of Han, Y. and Wu, L.P. [36] indicated that FDI has negative backward spillover effects and positive horizontal spillover effects on domestic agricultural firm innovation, and that firms with stronger absorptive capacity are more likely to benefit from FDI spillovers.

Therefore, for countries with different regions and different levels of economic development, FDI’s impact on innovation will vary. Furthermore, in the absence of other factors, its direct impact on China’s technological innovation ability is positive. The actual situation is far more complex than this. Therefore, this paper includes environmental pollution and economic growth in the research model and comprehensively considers the impact of FDI to improve research accuracy and more rationally guide the inflow of capital.

### 2.3. FDI’s Impact on Environmental Pollution

In recent years, China, as a major developing country, has been steadily continuing to open up, and its proportion of FDI has surpassed that of the United States. Indeed, FDI inflows can stimulate economic development and may bring advanced technology and experience, but there may be a high environmental cost incurred in the course of such economic development. For China, which is in a critical period of transformation, will the continuous increase in FDI increase the country’s environmental burden? Does it contribute to environmental pollution? These concerns constitute global problems. The existing studies show that FDI’s impact on environmental pollution is uncertain, and the difference in the macroeconomic environment affects FDI’s impact on environmental pollution, thus yielding uncertain results. FDI’s effects on the host country’s pollution include several results: the pollution paradise effect, the pollution halo effect, and the nonlinear relationship.

Omri and Kahouli [7] studied the correlation between energy consumption and FDI by establishing a panel data model for 1990–2011 on 65 countries. The results showed that there was a positive causal relationship between FDI and energy consumption in all countries except low-income countries, indicating that an increase in FDI would lead to an increase in energy consumption, which would probably lead to the emission of more pollutants. Tang and Tan [9] used a cointegration model and a causality model to study the influence of energy consumption and FDI on carbon dioxide emissions in Vietnam, concluding that FDI was one of the main factors causing Vietnam’s carbon dioxide emissions. Liu et al. [37] considered panel data on 112 Chinese cities and performed a comparative study of the impact of domestic investment and FDI on the environment. The results showed that both investment types had negative impacts on the environment; however, the degrees of influence were different.

Simultaneously, many scholars have verified FDI’s pollution halo effect on the host country’s environment. For example, Paramati et al. [38] used a causal relationship model to study panel data on 20 emerging market economies worldwide. The empirical results showed that FDI inflows had a significant positive impact on a country’s clean energy consumption, which was conducive to improving the environmental quality of host countries to some extent. Lee [22] studied the contribution of FDI inflows to clean energy use and carbon dioxide emissions in G20 countries and analyzed the long-term equilibrium relationship among variables using a cointegration model, observing that FDI inhibited carbon dioxide emissions of the economy and played an important role in realizing the green growth target. Abdouli and Hammami [39] explored the causal relationship between environmental quality and FDI in Middle Eastern and North African (MENA) countries using a simultaneous-equation panel data vector autoregression (VAR) model, concluding that FDI could reduce industrial pollution in the studied countries and that there was a pollution halo effect. Yue et al. [26] performed an empirical study using panel data on 104 Chinese cities based on the SBM-DDF model. Wei et al. [40] used the multi-Stochastic frontier model (SFA) model and a modified Stochastic Impacts by Regression on Population, Affluence, and Technology (STRIPAT) model to investigate the impact of FDI on the total factor energy efficiency (TFEE) of China’s manufacturing and found that FDI spillovers showed an overall increase across the period. It implied that FDI can save energy and reduce emissions through the spillover effect. The results showed that, overall, FDI had a positive impact on the green development of Chinese cities; however, the impact efficiency varied with industry and location.

In addition, some studies show that FDI’s impact on environmental pollution is a “conditional” effect or there is a nonlinear relationship between them. Zugravu-Soilita [41] indicated that FDI could reduce pollution in countries with a low average capital ratio and stricter environmental regulations. In contrast, FDI inflows could lead to pollution havens, albeit on a smaller scale, in countries that are well-capitalized and have loose environmental controls. Shahbaz et al. [42] apply the GMM method to examine the association between FDI and carbon emissions for the Middle East and North African (MENA) region, and they found there is an N-shaped association between FDI and carbon emissions. Liu et al. [37] studied the dynamic spatial clustering effect of FDI and various types of pollution in 285 Chinese cities and observed that FDI and the aggregation areas of environmental pollution were not completely consistent, which indicated that an increase in FDI might not lead to worsening pollution from a geographical perspective. The scholar further used a spatial panel model for analysis and concluded that FDI had a significant influence on various pollution categories. However, the influence direction was either positive or negative: its effect on dust pollution was negative, while the effect on wastewater discharge and SO_2_ pollution was positive.

## 3. Empirical Strategy and Data

### 3.1. Methodology

#### 3.1.1. Econometric Modeling

In this paper, we will establish three equations for the following quantities: economic growth, technological innovation, and environmental pollution.

(1) Economic Growth Equation.

The economic growth equation is established by using the Cobb-Douglas production function. The latter is a production function of the relationship between input and output, which was jointly studied by American mathematician Charles Cobb and economist Paul Douglas. The basic form of this function can be formulated as follows:

(1) $$Y=A\left(t\right)L^{\alpha }K^{\beta }\mu$$

where Y, $A\left(t\right)$, L, and K represent the total output value, the comprehensive technical level, the labor force input, and the capital input, respectively, while α and β represent the elasticities of labor output and capital output, respectively. Variable μ represents the random disturbance term.

To meet the needs of our research, we extend the C-D function to the following Equation (2), combining the endogenous growth theory, and the studies of Abdouli and Hammami [39] and Omri and Kahouli [7]:

(2) $$Y=A\left(t\right)L^{\alpha }K^{\beta }{\left(FDI\right)}^{\psi }En^{\lambda }$$

According to the studies by Jerzmanowski [14], Zhang and Liu [15], Klenow and Rodriguez-Clare [16], the comprehensive technical level $A\left(t\right)$ can be decomposed in several ways. Zhang and Liu [15] decompose it as $A\left(t\right)=Be^{at}F^{\gamma }C^{\theta }$, where B, F, and C represent, respectively, the exogenous technical change, the technical changes brought by the spillover effect, and the competition effect of FDI. Jerzmanowski [14], and Klenow and Rodriguez-Clare [16] put forward an assumption that the total factor productivity is determined by both the available technology (denoted by $T_{i}$) and efficiency with which such technology is used (denoted by $E_{i}$), and is expressed as $A_{i}=T_{i}E_{i}$. Here, we follow the approach proposed by Jerzmanowski [12] and Klenow and Rodriguez-Clare [16] to decompose $A\left(t\right)$. The researchers’ explanation meets our need that a portion of $A\left(t\right)$ differences be caused by their relations with factors.

Therefore, we extend the C-D function as follows:

(3) $$Y=ET^{\theta }L^{\alpha }K^{\beta }{\left(FDI\right)}^{\psi }En^{\lambda }$$

where Y is the real GDP, T represents the available technology, and E denotes the efficiency of the use of this technology. Variables L, K and FDI represent labor, capital stock, and foreign direct investment flow, respectively. To consider environmental pollution, an energy consumption variable En has been added here. θ, α, β, ψ, and λ are production elasticities.

To some extent, there is a linear relationship between energy consumption (En) and pollution (R): En=b×R. Therefore, Equation (3) can be transformed into the following:

(4) $$Y=ET^{\theta }L^{\alpha }K^{\beta }{\left(FDI\right)}^{\psi }{\left(bR\right)}^{\lambda }$$

where we assume that there is a relationship among θ, α, β, λ and ψ: θ+α+β+ψ+λ=1. A large proportion of China’s wastewater, waste gas, and dust are generated by consumption of resources in industrial production, especially in the context of energy use. Therefore, R can represent SO_2_ emissions, the total wastewater discharge, COD emissions, or ammonia nitrogen emissions. After dividing Equation (4) by L, we obtain:

(5) $$\frac{Y}{L}=Eb^{\lambda }{\left(\frac{T}{L}\right)}^{\theta }{\left(\frac{K}{L}\right)}^{\beta }{\left(\frac{L}{L}\right)}^{\alpha }{\left(\frac{FDI}{L}\right)}^{\psi }{\left(\frac{R}{L}\right)}^{\lambda }$$

Then, a log-linear regression panel data model of Equation (5) can be proposed. Below, provinces are indexed by subscript *i* (*i = 1, …, N*), while *t* represents the time period (*t = 1, …, T*).

(6) $$\ln{\left(\frac{Y}{L}\right)}_{it}=\alpha_{0}+\alpha_{1}ln{\left(\frac{T}{L}\right)}_{it}+\alpha_{2}\ln{\left(\frac{K}{L}\right)}_{it}+\alpha_{3}\ln{\left(\frac{FDI}{L}\right)}_{it}+\alpha_{4}\ln{\left(\frac{R}{L}\right)}_{it}+\alpha_{5}Z_{it}+d_{i}+v_{t}+\mu_{it}$$

where $\alpha_{5}$ represents the coefficient of control variables $Z_{it}$ that includes three-way FDI spillover: forward (denoted by fs), backward (denoted by bs), and horizontal (denoted by hs); the calculations of the three spillover indexes are shown in Appendix A. Variables $d_{i}$, $v_{t}$, $\mu_{it}$ represent, respectively, time effects, country-specific effects and the error term. The rationale for this model is that FDI brings into a host country not only capital but also most importantly a package of intangible assets. As a result, FDI can contribute to a host country’s economic growth not only through direct capital input but also through spillover effects in the form of increased productivity of the host country’s domestic firms as stated by Javorcik [17]. Some studies have shown that financial development is also closely related to economic growth, ecological environment, and technological innovation (Wang et al. [43]; Shahnazi and Shabani, [44]; Umar et al. [45], Barrell and Nahhas, [46]).Thus, we add three-way FDI spillover variables (fs*,* bs*,* and hs), financial development (fd, $fd^{2}$), and the openness to trade (open) to the control variables’ list to research the channels of FDI’s effects on economic growth, technology, and pollution emission.

(2) Technological Innovation Equation.

In studying FDI’s impact on technological innovation in China’s provinces, this paper combines many scholars’ results and presumes that innovation can be regarded as the output process of new knowledge [47,48,49]. Therefore, our empirical analysis considers the technological innovation equation of the following basic functional form according to the studies of Hu [50], Abdouli and Hammami [39], and Crescenzi et al. [51]:

(7) $$T_{it}=f\left(GDP_{it},\;R\&D_{it},\;Hum_{it},\;FDI_{it}\right)$$

where T represents technological innovation, and GDP, R&D, Hum, and FDI represent, respectively, the gross domestic product, the research and development expenditures, the number of employees in the science and technology-oriented industries, and foreign direct investment.

For this study, the actually used model of technological innovation is transformed into the following log-linear regression form:

(8) $$lnT_{it}=\beta_{0}+\beta_{1}\ln{\left(Hum\right)}_{it}+\beta_{2}\ln{\left(GDP\right)}_{it}+\beta_{3}\ln{\left(R\&D\right)}_{it}+\beta_{4}\ln{\left(FDI\right)}_{it}+\beta_{5}X_{it}+d_{i}+v_{t}+\mu_{it}$$

where $\beta_{5}$ represents the coefficient of control variables $X_{it}$ that includes financial development (fd, $fd^{2}$), the openness to trade (open), and three-way FDI spillover: forward (denoted by fs), backward (denoted by bs), horizontal (denoted by hs); the calculations of the three spillover indexes are shown in Appendix A. Pollution R could also be included in $X_{it}$, but the effect of R on T is uncertain. We expect pollution to have a positive effect on technological innovation. Moreover, we believe that the increase in environmental pollution will inevitably lead to people’s dissatisfaction, which in turn will stimulate enterprises to improve technical capabilities. Finally, in the above model $d_{i}$, $v_{t},$ and $\mu_{it}$ represent, respectively, time effects, country-specific effects and the error term.

(3) Environmental Pollution Equation.

Combining the research models of Shahbaz et al. [52], Liu et al. [37], Zugravu-Soilita [41], Yue et al. [26], and Tang and Tan [9], we observe that environmental pollution can be affected by regional economic growth, energy consumption, FDI, technological innovation capacity, and the environmental regulations’ strictness. Based on the above theoretical findings, we specify the general pollution emission function of the following form:

(9) $$R_{it}=f\left(GDP_{it},\;T_{it},\;En_{it},\;FDI_{it},\;ER_{it}\right)$$

where R, T, GDP, En, and FDI represent, respectively, pollution emissions, technological innovation, the gross domestic product, energy consumption and foreign direct investment, and ER reflects environmental regulations’ strictness. We transform the function into the natural logarithmic form to use a log-linear specification in order to keep the form consistent with the above two models, and it is agreed that this form (in contrast to the linear case) can lead to consistent and reliable empirical results.

(10) $$lnR_{it}=\varphi_{0}+\varphi_{1}\ln GDP_{it}+\varphi_{2}\ln FDI_{it}+\varphi_{3}\ln ER_{it}+\varphi_{4}lnEn_{it}+\varphi_{5}lnT_{it}+\varphi_{6}lnM_{it}+d_{i}+v_{t}+\mu_{it}$$

In Equation (10), R represents pollution emissions. In this paper, pollution includes SO_2_ emissions ($SO_{2}$), the total wastewater discharge (ww), chemical oxygen demand emissions (choe), and ammonia nitrogen emissions (nhe). M represents control variables, including urban population density (Pop), financial development (fd, $fd^{2}$), the openness to trade (open) and the three-way FDI spillover, namely, forward (denoted by fs), backward (denoted by bs), and horizontal spillover (denoted by hs).

#### 3.1.2. Dynamic Panel Simultaneous Equation Model

In addition, this paper uses a simultaneous equation model to describe economic behavior, which was first studied by Samuelson and Hicks. In reality, some economic phenomena often have complex mutual influence relations, and single-equation models have been insufficient for solving problems. Therefore, simultaneous equation models, which can more accurately describe the comprehensive interaction between variables, have gradually been adopted by many studies. After analyzing previous studies, we observe that there may be complex interactions among economic growth, technological innovation, environmental pollution, and FDI. In studying FDI’s impact on the other three factors, we adopt a simultaneous equation model and include these four factors in the same system to obtain more accurate experimental results.

As the data type we use is panel data and the one-period lagged levels of endogenous variables (i.e., GDP, technological innovation, and pollution emissions) can affect their current levels [7,53], we allow our simultaneous-equation models to have a dynamic panel specification. Our dynamic panel simultaneous-equation models are then estimated by using the generalized method of moments estimator. This approach uses a set of instrumental variables to solve the endogeneity problem of the regressors.

The specific form of the dynamic panel simultaneous-equation models used in this paper is defined in the following Equations (11)–(13) that follow from Equations (6), (8) and (10):

(11) $$\ln{\left(y\right)}_{it}=\alpha_{0}+\alpha_{1}ln{\left(y\right)}_{it-1}+\alpha_{2}\ln k_{it}+\alpha_{3}\ln fdi_{it}+\alpha_{4}\ln T_{it}+\alpha_{5}lnR_{it}+\alpha_{6}Z_{it}+v_{i}+\mu_{it}$$

(12) $$lnT_{it}=\beta_{0}+\beta_{1}\ln T_{it-1}+\beta_{1}\ln{\left(Hum\right)}_{it}+\beta_{2}\ln y_{it}+\beta_{3}\ln{\left(RD\right)}_{it}+\beta_{4}\ln{\left(fdi\right)}_{it}+\beta_{5}X_{it}+v_{i}+\mu_{it}$$

(13) $$lnR_{it}=\varphi_{0}+\varphi_{1}\ln R_{it-1}+\varphi_{1}\ln y_{it}+\varphi_{2}\ln fdi_{it}+\varphi_{3}\ln ER_{it}+\varphi_{4}lnEn_{it}+\varphi_{5}lnT_{it}+\varphi_{6}lnM_{it}+v_{i}+\mu_{it}$$

In Equation (11), y represents economic growth, and fdi*,* T*,* R represent foreign direct investment flow, technological innovation, and pollution emissions, respectively. Control variables $Z_{it}$ include financial development (fd, $fd^{2}$), the openness to trade (open), and three-way FDI spillover: fs*,* bs*,* and hs. In Equation (12), T, y*,* and fdi have the same meanings as in Equation (11). Hum and RD represent, respectively, the number of employees in the science and technology-oriented industries and the research and development expenditures. Control variables X include financial development (fd, $fd^{2}$), the openness to trade (open), and three-way FDI spillover (fs*,* bs*,* and hs) and $\ln{\left(y\right)}_{it-1}$. In Equation (13), control variables M include financial development, the openness to trade, urban population density (Pop), $\ln{\left(y\right)}_{it-1}$, fs*,* bs*,* and hs.

### 3.2. Estimation Method

There are two methods of estimating a simultaneous equation system: single-equation estimation and system estimation. The latter uses all information of the system to estimate all structural equations at the same time, and obtain parameter estimates of all equations simultaneously. This paper uses this method. Possible implementations of the system estimation method can follow the three-stage least method (3SLS), the full information maximum likelihood method (FIML), or the generalized method of moments. Here we focus on the latter.

Our estimated model is a dynamic model, and the time series observations in our sample are thirteen years. The researches of Blundell and Bond [54], and Barrell and Nahhas [46] show that the system’s GMM estimation can effectively overcome the problem of weak instrumental variables, greatly improve the finite sample performance of the estimator, and improve the accuracy of the estimation while reducing the problem of downward bias of the autoregressive coefficients. So, it is best to use a systems GMM approach to estimate our model. GMM is a parameter estimation method that requires that the actual parameters of the model satisfy some moment conditions. It is a generalization of the moment estimation method that allows random error terms to be heteroscedastic and serially correlated, and is more widely used than other traditional estimation methods. The idea of the GMM estimation method is to define the criterion function $Q\left(∆\right)$ as the correlation function between the instrumental variable and the disturbance term, and minimize the function to obtain the estimated values of parameters:

$$Q\left(∆\right)=u^{\prime }Z{\hat{\Omega }}^{-1}Z^{\prime }u$$

where ∆ denotes the parameters we estimate. Variables u, Z, and Ω are the residual vector, instrumental variable, and covariance matrix estimator of sample moments, respectively.

### 3.3. Data and Descriptive Statistics

#### 3.3.1. Data

The data type considered in this study is panel data, which refers to data tracking the same group of individuals within a period of time, including both the cross-sectional and time dimensions. The three targets of this study are economic growth, technological innovation, and environmental pollution. The selected data are annual Chinese provincial data from 2004 to 2016. Due to a significant data loss in Tibet and the different institutional environments in Hong Kong, Taiwan, and Macao, only 30 provinces, cities, and autonomous regions are considered in this paper. Tibet, Hong Kong, Taiwan, and Macao are outside the research scope. In addition, the data we have used is balance panel data, and there are no missing observations in our sample range. Data are collected from the China Statistical Yearbook, the China Industrial Statistics Yearbook, and the China Trade and External Economic Statistics Yearbook.

In Equation (11), y represents economic growth, measured by the provincial GDP per capita (L), and L is defined as the employed population. Variables fdi*,* T, and R represent foreign direct investment flow, technological innovation and pollution emissions, respectively, and fdi is defined as the provincial investment of foreign enterprises per capita. T is calculated as the number of domestic patent grants per capita [55]. Variables y, fdi, and T have been CPI-adjusted to a constant price value based on 2004. R is defined as SO_2_ emissions per capita (denoted by $SO_{2}$). Alternatively, R may stand for the total wastewater discharge per capita (denoted by ww), chemical oxygen emissions per capita (denoted by choe), or ammonia and nitrogen emissions per capita (denoted by nhe). Control variables $Z_{it}$ include three-way FDI spillover (fs*,* bs*,* and hs), financial development (fd, $fd^{2}$) and the openness to trade (open). It is very appropriate to use economic freedom to reflect the financial development of a country. However, there is no index of economic freedom at the provincial level in China, and the legal environment and government environment are similar in China. Therefore, financial development is defined as the ratio of loans of the banking system to GDP (denoted by fd). The openness to trade is defined as the ratio of the sum of import and export to GDP (denoted by open). The calculations of the three spillover indexes are shown in Appendix A. Variable k represents the capital stock calculated using the perpetual inventory method (PIM):

$$k_{it}=\left(1-\delta \right)k_{it-1}+I_{it}$$

Following Zhang et al. [46], we choose the gross fixed capital formation in year t deflated by price index for fixed asset investment, denoted by $I_{it}$. Zhang et al. [46] set δ of 9.6%. As different fixed assets account for different proportions of all fixed assets in each region, we follow the method of Zhang et al. [56] to calculate δ, but the value of δ we used as following is not a constant. We select the method of Hall and Jones [57] to calculate the capital stock for the base period ($k_{0}$) in 2004.

$$\delta_{it}=\frac{FAIC_{it}\times 6.9\%+FAIME_{it}\times 14.9\%+FAIOT_{it}\times 12.1\%}{TFAI_{it}}$$

where FAIC, FAIME, FAIOT, and TFAI respectively represents fixed asset investment in construction, machinery and equipment, other types of fixed asset investment, and total fixed asset investment.

In Equation (12), the measurements of T, y, and fdi have been defined in Equation (11). Variables Hum and RD are measured, respectively, by the number of R&D personnel and corporate R&D expenditure. The latter is CPI-adjusted to a constant price value based on 2004. The R&D we have considered refers to the innovation process that uses knowledge gained from basic research, applied research, and practical experience to produce new products, establish new processes or services, and improve the mentioned aspects. Variable X includes financial development (fd, $fd^{2}$) and the openness to trade (open) and three-way FDI spillover (s*,* bs*,* and hs)).

In Equation (13), M includes fd*,* $fd^{2}$*,* open*,* the urban population density (Pop), fs, bs, and hs. Variables Pop and En are calculated as the ratio of provincial population to land area, and the total consumption of various types of fossil energy per capita, respectively. ER is calculated according to the following formula:

(14) $$ER_{it}=\frac{nPuI_{it}}{IAV_{it}\sum_{t=1}^{T}\frac{PuI_{it}}{IAV_{it}}}\;\left(i=1,2\ldots 30;t=1,2,\ldots 12\right)$$

where ER, PuI, and IAV represent the environmental regulation index, the amount invested in industrial pollution control, and the industrial added value. Furthermore, cities are indexed by subscript $i\;\left(i\;=\;1,\;...,\;N\right)$, while t represents the time period $\left(t\;=\;1,\;\ldots ,\;T\right)$.

#### 3.3.2. Descriptive Statistics

As shown in Table 1, n = 30 and t = 13; thus, the data type is short panel data. To eliminate partial heteroscedasticity and make the data more robust, we use the logarithm of each variable except the ratio variable. The statistical results for each variable are as follows.

Descriptive statistics of various variables are presented in Table 1. On average, regions with the highest GDP include Beijing, Shanghai, Tianjin, and Jiangsu. Among the regions with the lowest GDP are Gansu, Yunnan, Shanxi, and Sichuan, most of which are in western China. In particular, Shanxi is a major coal-producing province.

The most densely distributed regions of patent grants are Guangdong, Jiangsu, and Zhejiang, where manufacturing and high-tech industries are concentrated. Regions with the lowest number of patent grants include Qinghai, Ningxia, and Hainan.

There are four variables that characterize pollution. The data shows that SO_2_ emissions tend to rise gradually and level off. Overall, ammonia and nitrogen emissions have followed a downward trend. Emissions of chemical oxygen have exhibited a volatile decline, especially in recent years.

## 4. Empirical Results and Discussion

### 4.1. Panel Unit Root Test

If there are random trends or structural changes in a dataset, data may be nonstationary. Results of a direct regression estimation of a model with nonstationary data will not be economically significant, and the estimated parameters will be biased, which will lead to the invalidation of other tests and ultimately cause the problem of “false regression”. Therefore, we first test the stationarity of data. Here, we use the unit root test method for panel data. To some extent, the panel data’s unit root test results are more convincing than analyses of the time series data itself.

Table 2 shows for each variable the results of two types of panel unit root test with their respective test characteristics. The unit root test for each variable has at least one result that rejects the null hypothesis. Thus, we can be confident that the logarithmic variables are stable.

### 4.2. Hausman Test

In general, there are three strategies for estimating panel data: mixed regression, fixed-effect models, and random-effects models. Here, the Hausman test is performed for each equation to select the appropriate regression model.

Table 3 shows the results of the Hausman tests. The *p*-values of such tests of economic growth, technological innovation, SO_2_ emissions, COD discharge, ammonia nitrogen discharge model, and wastewater discharge models are all less than 0.05. Therefore, we reject the null hypothesis and use the fixed-effect model to estimate these quantities’ models. Theoretically, for the estimation of the variance of individual residuals, the estimation results of the random-effects model are consistent only when N (the number of individuals) approaches infinity. Fixed-effects models, however, reduce endogeneity by controlling for unobserved individual heterogeneity that does not change over time. We only have 30 individuals, so the fixed-effect model is more appropriate.

### 4.3. Overidentification Test

Assume that the model contains all exogenous and endogenous variables and that the residual obtained using the Instrumental Variable (IV) method is an auxiliary regression for all exogenous variables.

The estimation method adopted in this paper is GMM. Three specifications that correspond to Equations (11)–(13) are simultaneously estimated. Table 4, Table 5, Table 6, Table 7 report the results of diagnostic tests (the J-statistic for overidentification, and the AR(2) test for the existence of the second-order autocorrelation in first differences). The purpose of the J-statistic is to test the validity of overidentifying restrictions under the null hypothesis that such restrictions are obeyed.

To improve the identifiability of the model, all variables except endogenous ones are regarded as instrumental variables. In particular, FDI is a variable that is prone to having endogeneity, so it is necessary to select an instrumental variable to replace it to weaken endogeneity. The method used in this paper is to select the one-lag FDI as a special instrumental variable. All test statistics in Table 4, Table 5, Table 6, Table 7 indicate good statistical performance.

### 4.4. GMM Estimation Results of the Simultaneous Equation Model and Discussion

As the use of single-equation estimation will ignore the relationship between each equation and the equation disturbance term, which will cause deviation of the experimental results to some extent, we further adopt a more efficient system estimation method that considers all equations together and incorporates them into a system for research.

Table 4 shows the GMM estimation results of panel simultaneous equations of Equations (11)–(13), where R represents emission of SO_2_. The overall findings show evidence of bidirectional causal links between economic growth and technology innovation and between the latter and SO_2_ emissions, while the relationship between economic growth and pollution emissions is not significant. In addition, the results show that FDI has a positive and direct impact on economic growth and technological innovation, while its direct impact on SO_2_ emissions is negative, indicating that FDI inflows can promote regional economic growth in China and enhance its technological innovation capabilities. The findings are in line with the research provided by Aust et al. [58] which indicated that FDI increases the probability of better trends for African sustainable development goals. Overall, FDI can ease the pressure of pollution emissions (similar to the results provided by Jiang et al. [59]), which indicates that FDI’s effect on pollution is the pollution halo effect in China. The one-lag values of GDP, FDI, and SO_2_ emissions have a significant positive impact on their current values, indicating that these variables tend to increase over time. Omri and Kahouli [7] also observe the lagged values of GDP have a significant and positive impact on their current values.

More precisely, Specification 1 in Table 4 indicates that at the 1% confidence level, technological innovation has a significantly positive impact on economic growth, with 1 unit of technological innovation output leading to an increase in economic growth rate of 0.147 units. Our empirical evidence is consistent with the results reported by Fan et al. [60] for the mining industry, and this also further confirms the correctness of national policies for promoting innovative and high-quality development. The effect of SO_2_ emissions on economic growth is not obvious, but the significantly positive effect of capital stock on economic growth still exists. FDI inflows have a significant positive impact on economic growth, which is consistent with the results obtained by Lee [22], Omri and Kahouli [7], Hong [24], Yue et al. [26], Peng et al. [25], Gutiérrez-Portilla et al. [61], and Doğan et al. [62]. The results show that a 1% increase in FDI inflows increases regional GDP growth by approximately 0.097%. Moreover, considering the performance of control variables, we conclude that FDI influences economic growth through backward spillover, which significantly improves the economic growth rate. There is a nonlinear relationship between financial development and economic growth. Higher openness to trade has a significant positive impact on economic growth.

Specification 2 in Table 4 shows that both the current GDP and the one-lagged GDP have significant impacts on technological innovation. The difference is that the one-lagged GDP has a significantly positive impact on the output of technological innovation, which indicates that there is a delay in the impact of economic growth on technological innovation. In contrast, SO_2_ emissions have a significant negative impact on technological innovation. The direct impact of FDI on technological innovation output is positive. Erdal and Göçer [31], Zhang [32], Wang and Wu [33], and Yang et al. [63] have obtained similar results. However, our results were not statistically significant. The forward and backward spillover effects of FDI have a significant negative impact on technological innovation at the confidence levels of 1%. The forward spillover effect of FDI has a negative influence on innovation output. For a 1% increase in the forward spillover benefit of FDI, innovation output will decrease by 0.520%. However, for a 1% increase in the backward spillover effect of FDI, innovation outputs decrease by 0.011%. This indicates that foreign companies have a strong incentive to prevent the leakage of technical knowledge to domestic upstream suppliers and downstream enterprises.

In Specification 3 in Table 4, the one-lagged GDP has a significant restraining effect on the emission of SO_2_. For a 1 unit increase of the one-lagged GDP, the emission of SO_2_ will decline by 0.346 units. Research reported by Xu et al. [64] shows that economic growth in the eastern region can reduce SO_2_ emissions, while Jiang et al. [59] find the urban economic levels can improve environmental quality in China. Technological innovation can reduce SO_2_ emissions significantly. The coefficient of FDI is significant and negative, indicating that FDI has a pollution halo effect directly (the results reported by Xu et al. [64] also show that the increases in FDI dependence will curb such discharge). However, FDI can significantly reduce SO_2_ emissions of host countries through the forward and backward spillover effect’s channel. This suggests that foreign enterprises can encourage domestic upstream firms to achieve cleaner production based on their demand and supply for high-quality products upstream and downstream of the industrial chain.

Table 5 shows the GMM estimation results of panel simultaneous equations of Equations (11)–(13), where *R* represents COD emissions (*choe*). In general, there is bidirectional causal links between economic growth and technological innovation, while there are unidirectional influence relationships between economic growth and COD emissions, technological innovation, and COD emissions. The one-lag values of GDP, FDI inflow, and COD emissions have a significantly positive impact on their current values, indicating that these variables tend to increase over time. FDI has a positive and significant impact on China’s economic growth and technological innovation, and its significant impact on economic growth is consistent with the results obtained by Lee [22], Hong [24], Erdal and Göçer [31], Peng et al. [25], Yue et al. [26], Wang and Wu [33], Zhang [32], and Aust et al. [58]. Additionally, FDI has a negative and significant effect on COD emissions.

The coefficients for Specifications 1 and 2 in Table 5 differ only slightly numerically from those in Table 4, while the coefficient symbols are almost consistent. However, due to R being defined as COD emissions, the results shown for Specification 3 in Table 5 are quite different from those in Table 4. Results for Specification 1 show that the direct impact of FDI on economic growth is significantly positive [7,20,22,23,24,51,52]. For a 1% increase in FDI, GDP growth will increase by 0.09%. Specifically, FDI can significantly enhance economic growth through the backward spillover effect when the horizontal and forward spillover effects are not significant. An increase of 1% in the backward spillover effect of FDI can increase the GDP growth rate by 0.02%. Specification 2 shows that the direct impact of FDI on technological innovation output is positive, and Erdal and Göçer [31], Zhang [32], Wang and Wu [33], and Yang et al. [63] have obtained similar results. FDI can inhibit the output of technological innovation through the backward spillover effect, which still indicates that foreign enterprises strictly block the flow of their technical knowledge to upstream domestic suppliers.

In Specification 3, GDP can significantly increase COD emissions. The research provided by Bakhsh et al. [65] similarly shows that GDP is positively related with CO_2_ emissions. For a 1 unit increase of GDP, COD emissions will increase by 0.278 units. Notably, technological innovations can significantly reduce COD emissions. The results reported by Hashmi and Alam [66] and Mensah et al. [67] show that technology has an inhibitory effect on carbon dioxide emissions. For a 1% increase in technology innovation, COD emissions can be reduced by 0.069%. In addition, the horizontal and backward spillover effects of FDI can significantly increase COD emissions, while the forward effect can significantly restrain COD emissions.

Table 6 shows the GMM estimation results of panel simultaneous equations of Equations (11)–(13), where *R* represents ammonia and nitrogen discharge (*nhe*). Specifications 1 and 2 in Table 6 are only slightly different from the results in Table 5. However, due to *R* instead being defined as ammonia and nitrogen discharge, our results exhibit a significant difference in Specification 3, where GDP can significantly increase ammonia and nitrogen discharge (similar to the results of Bakhsh et al. [65]). For a 1 unit increase in GDP, ammonia and nitrogen emissions will increase by 0.429 units. On the other hand, technological innovation can reduce such emissions [66,67]. For a 1% increase in technological innovation, ammonia and nitrogen discharge will be reduced by 0.2%. The coefficient of FDI is not significant, indicating that FDI does not have a pollution haven effect or a pollution halo effect directly (similarly to the results reported by Shao et al. [68]). However, the backward spillover effect of FDI on ammonia and nitrogen discharge is significantly negative.

Table 7 shows the GMM estimation results of panel simultaneous equations of Equations (11)–(13), where *R* represents the total wastewater discharge (*ww*). In general, results for Specifications 1 and 2 in Table 6 are only slightly different from the results in Table 4 and Table 5. The one-lag values of GDP, FDI inflows, and the total wastewater discharge have a significant positive effect on the current value, which suggests that these variables tend to increase over time. FDI has a significant direct positive impact on regional GDP and technological innovation, but its indirect spillover effect inhibits such innovation’s output, which also shows that foreign companies strictly protect their own patented technologies. However, we observe that the forward and horizontal spillover effects of FDI inhibit economic growth, while the backward spillover effect of FDI has a positive effect on GDP growth.

In Specification 3, GDP can significantly increase the total wastewater discharge (similar to the results of Bakhsh et al. [65]). For a 1 unit increase in GDP, the total wastewater discharge will increase by 0.90 units. It is worth noting that technological innovation cannot reduce such discharge. Our empirical evidence is consistent with the results reported by Liu et al. [37], and Xu et al. [64]. Moreover, the inflow of FDI has a significant direct impact on such discharge. This result is consistent with Caglar [69]. Caglar’s [69] findings imply that FDI inflow induces environmental pollution in India and Morocco. For a 1 unit increase in FDI inflow, the amount of wastewater discharged will increase by 0.02 units. Our results are similar to those of Liu et al. [37]. Additionally, the research provided by Omri and Kahouli [7], Bakhsh et al. [65] show that FDI inflows have positive effects on CO2 emissions. Furthermore, we observe that FDI can significantly increase the total wastewater discharge through FDI’s backward spillover channel.

## 5. Conclusions

Although many scholars have studied the interactions among FDI, economic growth, technological innovation, and pollution, their studies explore only two or three of these factors. This paper studies all four simultaneously and includes them into the same system for research. Finally, we comprehensively analyze the influence of FDI on the other three factors. To make the sample set sufficiently large, this paper uses panel data for 2004–2016 on 30 province-level regions in China (except Tibet) as samples and constructs a dynamic panel simultaneous equation model for analysis. The aim is to clarify FDI’s impact on economic growth, technological innovation, and pollution to provide policymakers with reliable advice on how to guide foreign investment.

In general, there is a two-way causal relationship between economic growth and technological innovation, and an improvement of the latter can significantly enhance the former [50]. The positive impact of GDP on technological innovation output is delayed. Economic growth has a unidirectional influence on environmental pollution. The current economic growth will increase emissions of four types of pollutants. On the other hand, the relationship between technological innovation and these types of pollutants is more complicated. There are “benign feedback mechanisms” between technological innovation output, SO_2_ emission, and COD emissions, and between the former and ammonia and nitrogen discharge, i.e., an improvement of the technological innovation level can significantly reduce COD emissions and ammonia and nitrogen discharge. At the same time, a reduction of SO_2_ emission, COD emissions, and ammonia and nitrogen discharge will further feed back to the output of technological innovation, which will facilitate the improvement of technological innovation.

As to the impact of FDI, we observe that FDI has a positive and direct impact on economic growth [7,22,24,25,26,61], and it is statistically significant. Moreover, compared with the horizontal and forward spillover effects, FDI has a significantly positive pull effect on the domestic economy through the backward spillover effect [31,32,33]. FDI also has a positive and direct impact on the improvement of the regional technological innovation level [63]. However, the indirect spillover effect of FDI on innovation is not statistically significant [40], which reflects the foreign companies’ strict protection of their own technical knowledge. Finally, FDI can directly reduce SO_2_ and COD emissions while increasing the total wastewater discharge, and the channels through which FDI indirectly affects the discharge of the three pollutants are complicated. However, FDI effects ammonia and nitrogen discharge mainly through the indirect way.

Based on the results of our empirical analysis, we conclude that FDI has positive effects on economic growth and technological innovation in various regions of China. Therefore, relevant departments should continue to develop foreign investment policies, create a better investment environment, and guide foreign investment to serve China’s high-tech industries. As to environmental pollution, the inflow of FDI has a direct impact on the increase in regional wastewater discharge. However, the direct effect of FDI on the emission of SO_2_, COD is significantly negative, indicating that FDI has a pollution halo effect on them directly. Different treatment methods should be used for different pollution types. Our research results confirm that foreign direct investment and technological advancement can simultaneously reduce SO_2_ emissions and COD emissions. The possibility of reducing ammonia and nitrogen discharge by increasing FDI backward and forward spillover is confirmed by our study.

## Figures and Tables

**Table 1:** Summary statistics (for logarithms of variables) for the period of 2004–2016.

**Region**	Mean						
	y	T	$SO_{2}$	choe	nhe	ww	fdi
Anhui	143,591.7	25,492.23	73,046.92	81,186.39	9364.256	2.85 × 10^8^	27,338.31
Beijing	128,858.2	41,755.08	13,379.35	13,845.49	1577.978	1.38 × 10^8^	91,767.23
Chongqing	125,300.3	15,989.08	135,230.6	55,845.81	6469.876	2.84 × 10^8^	35,611.15
Fujian	151,079.1	24,449.62	49,198.12	57,397.37	6874.098	3.01 × 10^8^	94,407.55
Gansu	102,868.6	2913.308	164,197.8	74,232.02	9677.556	1.69 × 10^8^	8863.61
Guangdong	158,019.2	120,454.2	43,737.66	51,632.26	6030.769	3.18 × 10^8^	111,128.40
Guangxi	143,852.8	5438.769	139,233.9	167,336.6	12,803.32	4.89 × 10^8^	28,656.64
Guizhou	121,461.4	4894.692	340,797.5	75,858.87	7315.475	2.11 × 10^8^	11,057.20
Hainan	110,050.7	884.0769	16,795.3	80,413.61	7988.159	2.43 × 10^8^	166,396.30
Hebei	198,955.2	12,737.31	161,182.5	101,180.1	9299.005	3.15 × 10^8^	29,650.51
Heilongjiang	70,360.6	10,369	41,563.99	66,048.01	5308.232	1.10 × 10^8^	9031.56
Henan	274,182.3	20,170.15	179,903.2	126,126.3	14,729.47	4.99 × 10^8^	32,705.48
Hubei	135,996.1	18,211.23	67,598.41	76,809.29	9141.114	2.83 × 10^8^	28,447.68
Hunan	151,690.5	15,767.69	88,521.91	111,145.8	12,674.23	3.19 × 10^8^	20,955.36
Jiangsu	564,483.6	133,607.4	184,740.5	161,607.1	16,698.2	9.38 × 10^8^	484,146.00
Jiangxi	162,857.8	8358.692	111,863.9	112,577.2	10,942.53	3.42 × 10^8^	49,430.16
Jilin	35,740.34	4814	19,581.12	25,550.34	2049.409	5.74 × 10^7^	8825.36
Liaoning	148,223.6	15,444.62	103,718.5	77,042.65	7980.085	2.32 × 10^8^	85,641.86
Neimenggu	193,355.9	2574.308	293,909	97,689.9	8836.252	1.82 × 10^8^	29,342.30
Ningxia	122,551.5	948	323,721	143,899.9	11,425.14	3.34 × 10^8^	24,181.78
Qinghai	113,308.9	468.3077	145,071.3	88,527.95	8829.724	2.30 × 10^8^	17,707.86
Shandong	207,644.5	50,173.31	109,329.2	67,559.55	6627.785	2.58 × 10^8^	50,398.25
Shanghai	248,022.5	38,107.38	63,833.08	45,419.43	6578.039	3.96 × 10^8^	373,501.40
Shanxi1	90,361.62	5020.615	170,044.6	50,326.67	5963.292	1.51 × 10^8^	16,627.95
Shanxi2	152,295.8	14,028.38	150,212.1	70,744.39	7034.998	2.15 × 10^8^	25,965.57
Sichuan	136,180.3	29,475	102,660.6	90,622.58	8633.14	2.76 × 10^8^	25,841.29
Tianjin	233,505.8	15,585.62	68,876.19	49,730.98	5769.727	2.14 × 10^8^	193,205.00
Xinjiang	108,663.3	3273	154,475.1	98,882.75	7611.937	1.97 × 10^8^	6639.75
Yunnan	110,665.4	4912.154	100,098.2	66,222.77	5916.428	2.06 × 10^8^	17,905.00
Zhejiang	154,977.5	116,989.9	49,355.9	42,701.39	4973.814	2.58 × 10^8^	75,267.36

**Table 2:** Results of the panel unit root test.

Variable	LLC	HT	Stationarity	Variable	LLC	HT	Stationarity
lnT	−3.520 ***	2.186	Yes	lnchoe	−0.274	−3.874 ***	Yes
lnfdi	−3.842 ***	0.500	Yes	lnww	−1.816 **	−4.337 ***	Yes
lnk	−0.902	−9.638 ***	Yes	lny	0.302	0.363 ***	Yes
$lnSO_{2}$	−2.244 **	0.283 ***	Yes	lnPop	−40.698 ***	−2.493 ***	Yes
ER	−4.265 ***	−11.189 ***	Yes	lnRD	−1.042	−14.653 ***	Yes
lnnhe	−3.074 ***	−4.817 ***	Yes	lnHum	−2.341 ***	−12.081 ***	Yes
hs	−2.6214 ***	−15.6659 ***	Yes	lnEn	−5.9700 ***	−3.7577 ***	Yes
bs	−4.460 ***	−41.169 ***	Yes	fs	−4.649 ***	0.722	Yes
fd	−3.1260 ***	2.4120	Yes	open	−8.8420 ***	3.2327	Yes

Note: The null hypothesis of the Levin-Lin-Chu(LLC) and Harris-Tzavalis(HT) tests are that all panels contain a unit root. Significance levels are indicated as follows: *** *p* < 0.01, ** *p* < 0.05.

**Table 3:** Results of the Hausman test.

The Classification of R	Model	Chi^2^ Statistic	Prob.	Type of Model
$R=SO_{2}$	Model of economic growth	82.77 ***	0.000	Fixed effect
	Model of technological innovation	38.21 ***	0.000	Fixed effect
	Model of SO_2_ emissions	57.92 ***	0.000	Fixed effect
R=nhe	Model of economic growth	79.45 ***	0.000	Fixed effect
	Model of technological innovation	32.58 ***	0.002	Fixed effect
	Model of ammonia and nitrogen emissions	63.00 ***	0.000	Fixed effect
R=choe	Model of economic growth	78.96 ***	0.000	Fixed effect
	Model of technological innovation	31.17 ***	0.003	Fixed effect
	Model of COD discharge	61.02 ***	0.000	Fixed effect
R=ww	Model of economic growth	110.15 ***	0.000	Fixed effect
	Model of technological innovation	43.78 ***	0.000	Fixed effect
	Model of wastewater discharge	139.01 ***	0.000	Fixed effect

Note: The null hypothesis of the Hausman test is that $\mu_{i}$ is not related to $X_{it}$ and $z_{i}$, which means that the random-effect model is more suitable than other model types. Labels *** indicate significance at 10% levels, respectively.

**Table 4:** GMM estimation results of panel simultaneous equations of Equations (11)–(13) (R represents $SO_{2}$)

Specifications	Specification 1	Specification 2	Specification 3
Independent Variables	lny	lnT	$lnSO_{2}$
lny	-	−0.194 *** (0.040)	0.348 *** (0.029)
$lny\left(t-1\right)$	0.215 ***	0.281 ***	−0.346 ***
	(0.040)	(0.032)	(0.020)
lnT	0.147 ***	-	−0.021 ***
	(0.018)		(0.003)
$lnT\left(t-1\right)$	-	0.880 ***	-
		(0.022)	
$lnSO_{2}$	−0.009	−0.070 ***	-
	(0.030)	(0.014)	
$lnSO_{2}\left(-1\right)$	-	-	0.949 ***
			(0.007)
lnk	0.710 ***	-	-
	(0.036)		
lnfdi	0.097 ***	0.037	−0.021 **
	(0.021)	(0.026)	(0.008)
lnEn	-	-	0.029 **
			(0.011)
ER	-	-	0.009
			(0.005)
lnPop	-	-	0.006
			(0.007)
lnRD	-	0.044 **	-
		(0.022)	
lnHum	-	0.095 **	-
		(0.020)	
hs	−0.112	0.054	0.041
	(0.078)	(0.075)	(0.026)
fs	−0.296	−0.520 ***	−0.378 ***
	(0.182)	(0.185)	(0.063)
bs	0.017 ***	−0.011 ***	−0.378 ***
	(0.003)	(0.003)	(0.063)
fd	−0.203 ***	0.218 ***	0.128 ***
	(0.077)	(0.077)	(0.041)
$fd^{2}$	0.012	−0.058 **	−0.056 ***
	(0.024)	(0.024)	(0.015)
open	−0.151 ***	−0.038 **	0.028 **
	(0.031)	(0.221)	(0.016)
Constant	−3.188 ***	0.934	−0.142
	(0.745)	(0.295)	(0.139)
J-statistic (*p*-value)	28.462 (0.1609)	26.063 (0.1637)	26.852 (0.1395)
AR(2) test (*p*-value)	0.104 (0.9172)	0.303 (0.7617)	−0.623 (0.5333)

Note: Variable R is defined as the emission of SO_2_. J-statistic refers to the overidentification test for the restrictions in the GMM estimation. The AR(2) test is the Arellano-Bond test for the existence of the second-order autocorrelation in first differences. Labels ** and *** indicate significance at 5% and 10% levels, respectively. The use of $\left(t-1\right)\;$ means the lag of the variables.

**Table 5:** GMM estimation results of panel simultaneous equations of Equations (11)–(13) (R represents choe).

Specifications	Specification 1	Specification 2	Specification 3
Independent Variables	lny	lnT	lnchoe
lny	-	−0.212 ***	0.278 ***
		(0.035)	(0.049)
$lny\left(t-1\right)$	0.219 ***	0.248 ***	0.134 **
	(0.038)	(0.035)	(0.063)
lnT	0.139 ***	-	−0.069 ***
	(0.018)		(0.009)
$lnT\left(t-1\right)$	-	0.924 ***	-
		(0.017)	
lnchoe	0.003	−0.031	-
	(0.013)	(0.022)	
$lnchoe\left(t-1\right)$	-	-	0.703 ***
			(0.035)
lnk	0.714 ***	-	-
	(0.030)		
lnfdi	0.090 ***	0.034 *	−0.030 *
	(0.018)	(0.018)	(0.018)
lnEn	-	-	−0.108 ***
			(0.025)
ER	-	-	0.047
			(0.016)
lnPop	-	-	0.003
			(0.019)
lnRD	-	0.022	-
		(0.020)	
lnHum	-	0.068 ***	-
		(0.018)	
hs	−0.084	0.029	0.216 ***
	(0.072)	(0.071)	(0.051)
fs	−0.246	−0.205	−0.441 ***
	(0.199)	(0.125)	(0.143)
bs	0.018 ***	−0.018 ***	0.036 ***
	(0.003)	(0.004)	(0.014)
fd	−0.177 **	0.156 **	0.129
	(0.081)	(0.073)	(0.097)
$fd^{2}$	0.004	−0.038	−0.102 ***
	(0.026)	(0.024)	(0.033)
open	−0.154 ***	−0.070 ***	−0.094 ***
	(0.024$fd^{2}$	(0.023)	(0.036)
Constant	−3.173 ***	−0.569 **	1.211 ***
	(0.658)	(0.227)	(0.347)
J-statistic (*p*-value)	24.582 (0.372)	22.995 (0.289)	5.871 (0.997)
AR(2) test (*p*-value)	−0.191 (0.849)	0.725 (0.469)	−1.757 (0.079)

Note: Variable R is defined as the COD discharge. J-statistic refers to the overidentification test for the restrictions in the generalized method of moments (GMM) estimation. The AR(2) test is the Arellano–Bond test for the existence of the second-order autocorrelation in first differences. Labels *, ** and *** indicate significance at 1%, 5%, and 10% levels, respectively. The use of $\left(t-1\right)$ means the lag of the variables.

**Table 6:** Generalized method of moments (GMM) estimation results of panel simultaneous equations of Equations (11)–(13) (R represents nhe).

Specifications	Specification 1	Specification 2	Specification 3
Independent Variables	lny	lnT	lnnhe
lny	-	−0.211 ***	0.429 ***
		(0.036)	(0.110)
$lny\left(t-1\right)$	0.219 ***	0.246 ***	0.808 ***
	(0.040)	(0.032)	(0.066)
lnT	0.147 ***	-	−0.210 ***
	(0.018)		(0.025)
$lnT\left(t-1\right)$	-	0.921 ***	-
		(0.019)	
lnnhe	−0.019	−0.046 ***	-
	(0.013)	(0.018)	
$lnnhe\left(t-1\right)$	-	-	−0.290 ***
			(0.027)
lnk	0.729 ***	-	-
	(0.032)		
lnfdi	0.084 ***	0.039 *	0.032
	(0.019)	(0.022)	(0.071)
lnEn	-	-	−0.081
			(0.058)
ER	-	-	0.053
			(0.013)
lnPop	-	-	0.107 ***
			(0.025)
lnRD	-	0.030	-
		(0.022)	
lnHum	-	0.073 ***	-
		(0.019)	
hs	−0.090	0.067	0.231 ***
	(0.075)	(0.076)	(0.058)
fs	−0.231	−0.240	−0.583 **
	(0.204)	(0.147)	(0.286)
bs	0.016 ***	−0.018 ***	−0.085 ***
	(0.003)	(0.004)	(0.017)
fd	−0.223 ***	0.097	0.068
	(0.085)	(0.068)	(0.147)
$fd^{2}$	0.018	−0.019	−0.179 ***
	(0.027)	(0.023)	(0.048)
open	−0.139 ***	−0.086 ***	−0.453 ***
	(0.031)	(0.022)	(0.07)
Constant	−3.054 ***	−0.643 **	−0.733
	(0.040)	(0.036)	(0.910)
J-statistic(*p*-value)	27.50597 (0.193)	22.47347 (0.315)	23.574 (0.370)
AR(2) test (*p*-value)	−0.053089 (0.958)	0.111534 (0.911)	−0.000 (0.100)

Note: Variable R is defined as the ammonia nitrogen emissions. J-statistic refers to the overidentification test for the restrictions in the GMM estimation. The AR(2) test is the Arellano–Bond test for the existence of the second-order autocorrelation in first differences. Labels *, ** and *** indicate significance at 1%, 5%, and 10% levels, respectively. The use of $\left(t-1\right)$ means the lag of the variables.

**Table 7:** GMM estimation results of panel simultaneous equations of Equations (11)–(13) (R represents total wastewater discharge (ww*)*).

Specifications	Specification 1	Specification 2	Specification 3
Independent Variables	lny	lnT	lnww
lny	-	−0.272 ***	0.903 ***
		(0.043)	(0.012)
$lny\left(t-1\right)$	0.045 ***	0.187 ***	−0.838 ***
	(0.015)	(0.027)	(0.015)
lnT	0.038 ***	-	0.0006
	(0.011)		(0.002)
$lnT\left(t-1\right)$	-	0.909 ***	-
		(0.017)	
lnww	0.868 ***	0.084 ***	-
	(0.033)	(0.027)	
$lnww\;\left(t-1\right)$	-	-	0.900 ***
			(0.010)
lnk	0.127 ***	-	-
	(0.022)		
lnfdi	0.025 *	0.037 *	0.022 ***
	(0.013)	(0.019)	(0.005)
lnEn	-	-	−0.004
			(0.005)
ER	-	-	0.014 ***
			(0.003)
lnPop	-	-	0.018 ***
			(0.003)
lnRD	-	0.053 **	-
		(0.021)	
lnHum	-	0.099 ***	-
		(0.019)	
hs	−0.056 ***	0.004	0.013
	(0.019)	(0.083)	(0.019)
fs	−0.051	−0.198	−0.175 ***
	(0.084)	(0.132)	(0.038)
bs	0.002	−0.030 ***	0.010 ***
	(0.002)	(0.006)	(0.002)
fd	0.013	0.205 ***	−0.110 ***
	(0.029)	(0.078)	(0.020)
$fd^{2}$	−0.021 **	−0.036	0.035 ***
	(0.009)	(0.025)	(0.007)
open	0.071 ***	−0.061 **	−0.005
	(0.018)	(0.024)	(0.007)
Constant	−7.138 ***	−1.768 ***	0.872 ***
	(0.380)	(0.393)	(0.113)
J-statistic (*p*-value)	24.476 (0.435)	24.636 (0.216)	25.230 (0.193)
AR(2) test (*p*-value)	2.523 (0.012)	1.115 (0.265)	1.282 (0.200)

Note: Variable R is defined as the total wastewater discharge. J-statistic refers to the overidentification test for the restrictions in the GMM estimation. The AR(2) test is the Arellano–Bond test for the existence of the second-order autocorrelation in first differences. Labels *, ** and *** indicate significance at the 1%, 5%, and 10% levels, respectively. The use of $\left(t-1\right)$ means the lag of the variables.

## Data Availability

The data presented in this study are openly available in the China Statistical Yearbook, China Industrial Statistics Yearbook, China Trade and External Economic Statistics Yearbook, and statistical yearbooks of provinces and cities in China.

## Appendix A. Calculation of Three-Way FDI Spillover

### Appendix A.1. FDI’s Forward Spillover

Theoretically, the forward spillover effect of FDI represents a recognition that upstream foreign enterprises can influence downstream domestic firms by providing high-quality and high-tech inputs. Considering the availability of provincial data in China, we define the provincial forward FDI spillover effect as follows, consistently with Han and Wu [36] and Wei et al. [40]:

$$fs_{it}=\frac{SV_{it}^{f}-EV_{it}^{f}}{SV_{it}-EV_{it}}$$

where $fs_{it}$ represents the forward FDI spillover. $SV_{it}^{f}$ measures the revenue of foreign-funded industrial enterprises. $EV_{it}^{f}$ denotes the export revenue of foreign-funded enterprises. $SV_{it}$ and $EV_{it}$ represent, respectively, the industrial revenue, and the total export revenue in province i. The direct consumption coefficients at the provincial level are unavailable, and some input–output data are missing as well. Here, we use the ratio of foreign enterprises’ revenue in the host country ($SV_{it}^{f}-EV_{it}^{f}$) to the total domestic revenue of regional enterprises ($SV_{it}-EV_{it}$) to represent the forward FDI spillover. As before, provinces are indexed by subscript i (i = 1, …, N), while *t* represents the time period (*t* = 1, …, *T*).

### Appendix A.2. FDI’s Backward Spillover

Backward spillover reflects to a certain extent the demand of downstream foreign-funded enterprises for upstream intermediaries or services. As buyers, foreign-funded enterprises can improve the technical level of domestic suppliers by transferring technology. The formula for backward spillover, used by Han and Wu [36], Wei et al. [40], and Javorcik [17], is as follows:

$$bs_{it}=\frac{CO_{it}^{f}-IO_{it}^{f}}{CO_{it}-IO_{it}}$$

where $bs_{it}$ represents backward FDI spillover. $CO_{it}^{f}$ measures the main operating costs of foreign-funded enterprises. $IO_{it}^{f}$ denotes the import value of such enterprises. $CO_{it}$ and $IO_{it}$ represent, respectively, the total main operating cost in region i, and the total import value in province i. The direct consumption coefficients at the provincial level are unavailable, and some input–output data are missing as well. Here, we use the ratio of foreign enterprises’ revenue in the host country ($SV_{it}^{f}-EV_{it}^{f}$) to the total domestic revenue of regional enterprises ($SV_{it}-EV_{it}$) to represent the forward FDI spillover. As above, provinces are indexed by subscript i (i = 1, …, N), while *t* represents the time period (*t* = 1, …, *T*).

### Appendix A.3. FDI’s Horizontal Spillover

The horizontal spillover effect refers to FDI spillovers within the same industry, i.e., the phenomenon whereby domestic enterprises achieve technological progress by imitating the technology of foreign-funded enterprises or employing skilled workers. Considering the method proposed by Han and Wu [36], Wei et al. [40], and Javorcik [17] and taking into account the unavailability of the direct consumption coefficients at the provincial level in China, we calculate the horizontal spillover as follows:

$$hs_{it}=\frac{SV_{it}^{f}}{SV_{it}}$$

where $hs_{it}$ represents the horizontal FDI spillover. $SV_{it}^{f}$ and $SV_{it}$ measure, respectively, the revenue of foreign-funded industrial enterprises and the industrial revenue in region i. The direct consumption coefficients at the provincial level are unavailable, and some input–output data are missing. Here, we use the ratio defined above to represent the forward FDI spillover. As above, provinces are indexed by subscript i (i = 1, …, N), while *t* represents the time period (*t* = 1, …, *T*).
